# Applications and prospects of cryo-EM in drug discovery

**DOI:** 10.1186/s40779-023-00446-y

**Published:** 2023-03-06

**Authors:** Kong-Fu Zhu, Chuang Yuan, Yong-Ming Du, Kai-Lei Sun, Xiao-Kang Zhang, Horst Vogel, Xu-Dong Jia, Yuan-Zhu Gao, Qin-Fen Zhang, Da-Ping Wang, Hua-Wei Zhang

**Affiliations:** 1 Department of Biomedical Engineering, Southern University of Science and Technology, Shenzhen, 518055 Guangdong China; 2 Department of Biochemistry and Molecular Biology, School of Basic Medical Sciences, Peking University, Beijing, 100191 China; 3 Department of Structural Biology, St. Jude Children’s Research Hospital, Memphis, TN 38105 USA; 4 Center for Protein Science and Crystallography, School of Life Sciences, Faculty of Science, Chinese University of Hong Kong, Hong Kong, 999077 China; 5 Interdisciplinary Center for Brain Information, the Brain Cognition and Brain Disease Institute, Shenzhen Institute of Advanced Technology, Chinese Academy of Sciences, Shenzhen, 518055 Guangdong China; 6 Faculty of Life and Health Sciences, Shenzhen Institute of Advanced Technology, Chinese Academy of Sciences, Shenzhen, 518055 Guangdong China; 7 Shenzhen-Hong Kong Institute of Brain Science-Shenzhen Fundamental Research Institutions, Shenzhen, 518055 Guangdong China; 8 Shenzhen Institute of Advanced Technology, Chinese Academy of Sciences, Shenzhen, 518055 Guangdong China; 9 State Key Lab for Biocontrol, School of Life Sciences, Sun Yat-Sen University, Guangzhou, 510275 China; 10 Cryo-EM Facility Center, Southern University of Science and Technology, Shenzhen, 518055 Guangdong China; 11 Department of Orthopedics, Shenzhen Intelligent Orthopaedics and Biomedical Innovation Platform, Guangdong Provincial Research Center for Artificial Intelligence and Digital Orthopedic Technology, Shenzhen Second People’s Hospital, The First Affiliated Hospital of Shenzhen University, Shenzhen, 518000 Guangdong China; 12 Guangdong Provincial Key Laboratory of Advanced Biomaterials, Southern University of Science and Technology, Shenzhen, 518055 Guangdong China

**Keywords:** Cryo-electron microscopy (cryo-EM), Drug discovery, Structure-based drug design, Fragment-based drug discovery, Proteolysis targeting chimeras, Drug repurposing, Artificial intelligence (AI)

## Abstract

Drug discovery is a crucial part of human healthcare and has dramatically benefited human lifespan and life quality in recent centuries, however, it is usually time- and effort-consuming. Structural biology has been demonstrated as a powerful tool to accelerate drug development. Among different techniques, cryo-electron microscopy (cryo-EM) is emerging as the mainstream of structure determination of biomacromolecules in the past decade and has received increasing attention from the pharmaceutical industry. Although cryo-EM still has limitations in resolution, speed and throughput, a growing number of innovative drugs are being developed with the help of cryo-EM. Here, we aim to provide an overview of how cryo-EM techniques are applied to facilitate drug discovery. The development and typical workflow of cryo-EM technique will be briefly introduced, followed by its specific applications in structure-based drug design, fragment-based drug discovery, proteolysis targeting chimeras, antibody drug development and drug repurposing. Besides cryo-EM, drug discovery innovation usually involves other state-of-the-art techniques such as artificial intelligence (AI), which is increasingly active in diverse areas. The combination of cryo-EM and AI provides an opportunity to minimize limitations of cryo-EM such as automation, throughput and interpretation of medium-resolution maps, and tends to be the new direction of future development of cryo-EM. The rapid development of cryo-EM will make it as an indispensable part of modern drug discovery.

## Background

Drug discovery is closely related to human health. Early drug discovery and development mainly stemmed from accidental discovery and natural product screening [1]. There are many interesting examples, including the discovery of the antibacterial activity of penicillin and sulfonamides, and the large-scale screening of natural products to identify the antitumor drug paclitaxel and the antimalarial drug artemisinin [2–5]. Since the late twentieth century, breakthroughs in molecular biology, synthetic chemistry, structural biology, and computational techniques have brought great changes to the field of novel drug research and development [6]. The current drug design and discovery process is more directional and visible and requires a high degree of joint effort across multiple disciplines [7]. With further improvements in drug development strategies, new drug screening platforms have emerged based on traditional technologies. Presently, several of the most prevalent drug screening methods include the high-throughput screening of compound libraries and technologies such as structure-based drug design (SBDD), fragment-based drug discovery (FBDD), DNA-encoded chemical library, proteolysis targeting chimera (PROTAC) and drug repurposing, which account for an increasing proportion of contemporary drug development [8–12].

Structural biology has always played an important role in drug discovery since it provides the most direct and visible atomic-level information on drug targets, and it can be applied to every step of preclinical drug development, including the identification and design of drug targets and the optimization of lead compounds [13]. Based on atomic resolution information about the active or regulatory sites of target proteins, the structural design of drugs becomes practical. Presently, there are three predominant techniques for the study of structural biology, namely, X-ray crystallography, nuclear magnetic resonance (NMR), and cryogenic electron microscopy (cryo-EM) [14]. X-ray crystallography usually provides structural information at the atomic level, which has obvious advantages for crystallizable macromolecules [15]. However, X-ray crystallography mostly covers protein molecules and molecular complexes with a size of approximately 10–150 kD. As the yield of proper crystals becomes increasingly difficult with increasing molecular size, there are only a few crystal structures of target proteins beyond 150 kD and super-large protein complexes [16]. In addition, for membrane proteins, obtaining high-quality crystal structures is still full of uncertainties. On the other hand, without crystallization, NMR can directly analyze the structure of proteins in solution and provide valuable information about internal protein dynamics [17]. However, it requires the labelling of protein backbone and/or amino acid residues with NMR-sensitive isotopes and is limited to small proteins below 50 kD [18]. Cryo-EM is mainly used to analyze the structures of macromolecules larger than approximately 100 kD, and most cryo-EM structures with higher resolution than 3 Å are characterized as larger than 135 kD [16]. However, an increasing number of smaller proteins have been solved with the technical development of cryo-EM, such as Kirsten rat sarcoma virus, streptavidin, Lys-Asp-Glu-Leu receptor and receptor-binding domain of severe acute respiratory syndrome coronavirus 2 (SARS-CoV-2) spike protein [19–22]. Cryo-EM determines the structure of macromolecular complexes and membrane proteins in solution with relatively small amounts of protein and without crystallization [23]. In addition, since proteins are highly dynamic in solution close to their native state, time-resolved cryo-EM can capture multiple conformations during the reaction, which is advantageous for structure elucidation [24].

**Fig. 1:**
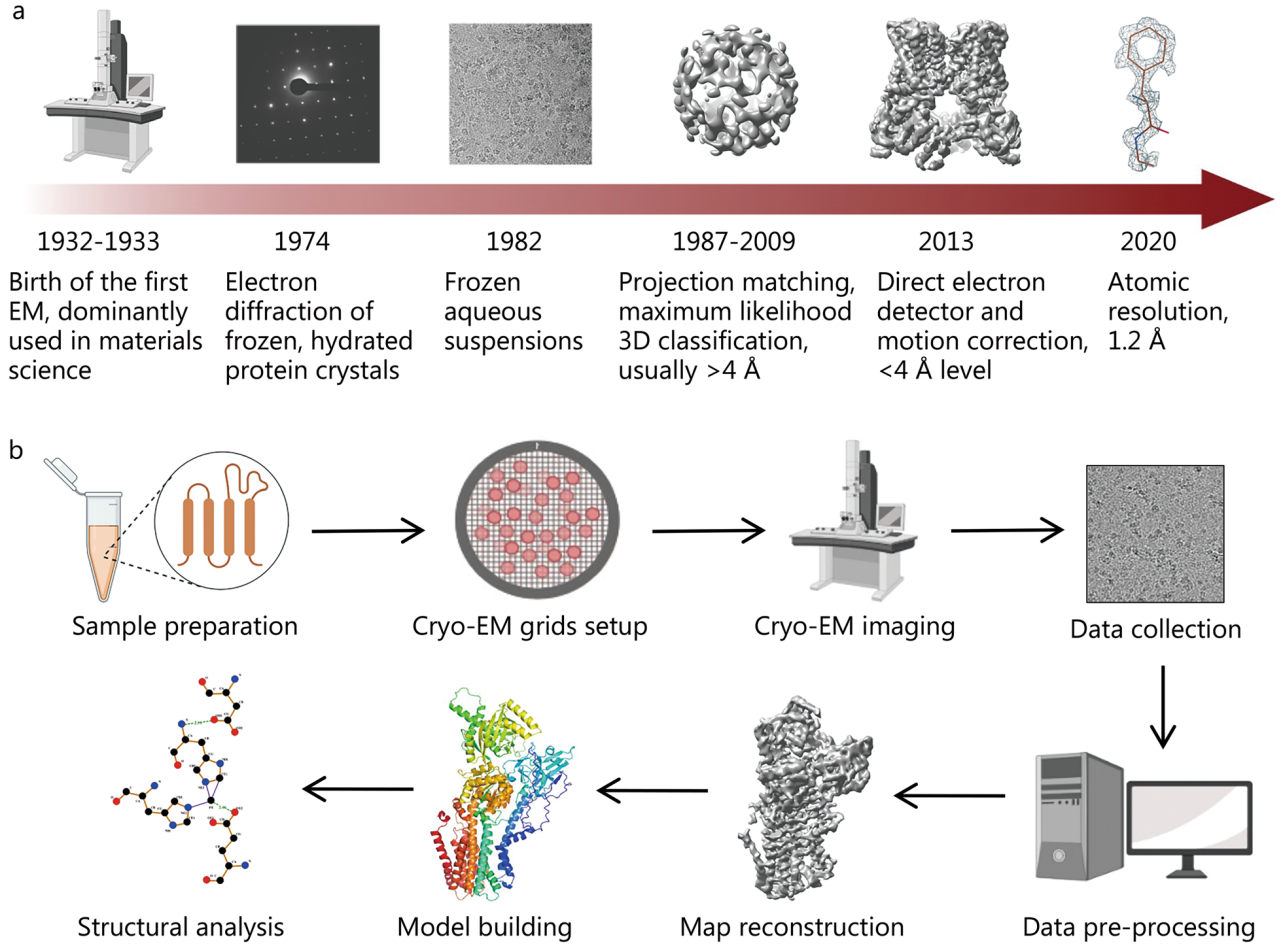
History and workflow of single particle cryo-electron microscopy (cryo-EM). **a** Key events in the development of single particle cryo-EM. **b** Typical workflow of single particle cryo-EM for structural analysis

The development of cryo-EM dates back to 1932, when the first electron microscopy was invented and predominantly used in materials science [25]. Thirty years later, the electron diffraction of protein crystals was first performed, which opened the door to the use of electron microscopy in the field of protein structure analysis [26]. With the development of a single-particle 3D reconstruction algorithm and the application of plunge-freezing techniques to biomacromolecules, cryo-EM has been gradually developed for structural biology since 1982 [27, 28]. However, it could hardly resolve structures within 4 Å before 2013. With the development of algorithms and hardware, especially the introduction of direct electron detectors, the resolution of cryo-EM has been greatly improved [29–32]. The Nobel Prize in Chemistry 2017 has been awarded to three pioneers (Jacques Dubochet, Joachim Frank, and Richard Henderson) for their development of cryo-EM techniques. In 2020 alone, 1753 cryo-EM structures within 4 Å were published in the Protein Data Bank [33]. To the best of our knowledge, the highest resolution of proteins analyzed by cryo-EM has reached 1.2 Å, which indicates that cryo-EM has truly reached atomic resolution (Fig. 1a) [23, 34, 35]. The cryo-EM procedure is shown in Fig. 1b, mainly including sample preparation, cryo-EM grids setup and imaging, data collection and pre-processing, and 3D map reconstruction as well as the subsequent model building and structure analysis. Despite the low resolution of early electron microscopy, cryo-EM has been used in combination with X-ray crystallography for drug development, including target identification and validation, since the 1990s [16]. In short, this approach did combine high-resolution crystal structure information with relatively low-resolution electron microscopic density maps, and through specialized adjustment and matching, it obtained information on difficult-to-crystallize macromolecular complexes. As cryo-EM has achieved atomic resolution, its role in the field of drug research and development has substantially increased in recent years. This review will illustrate how cryo-EM can facilitate the current drug discovery process through several examples in SBDD, FBDD, PROTAC, antibody drug development and drug repurposing. The combination of cryo-EM and other cutting-edge technologies such as artificial intelligence (AI) is also discussed, which may provide potential opportunities for further development of cryo-EM.

## Application of cryo-EM in SBDD

**Fig. 2:**
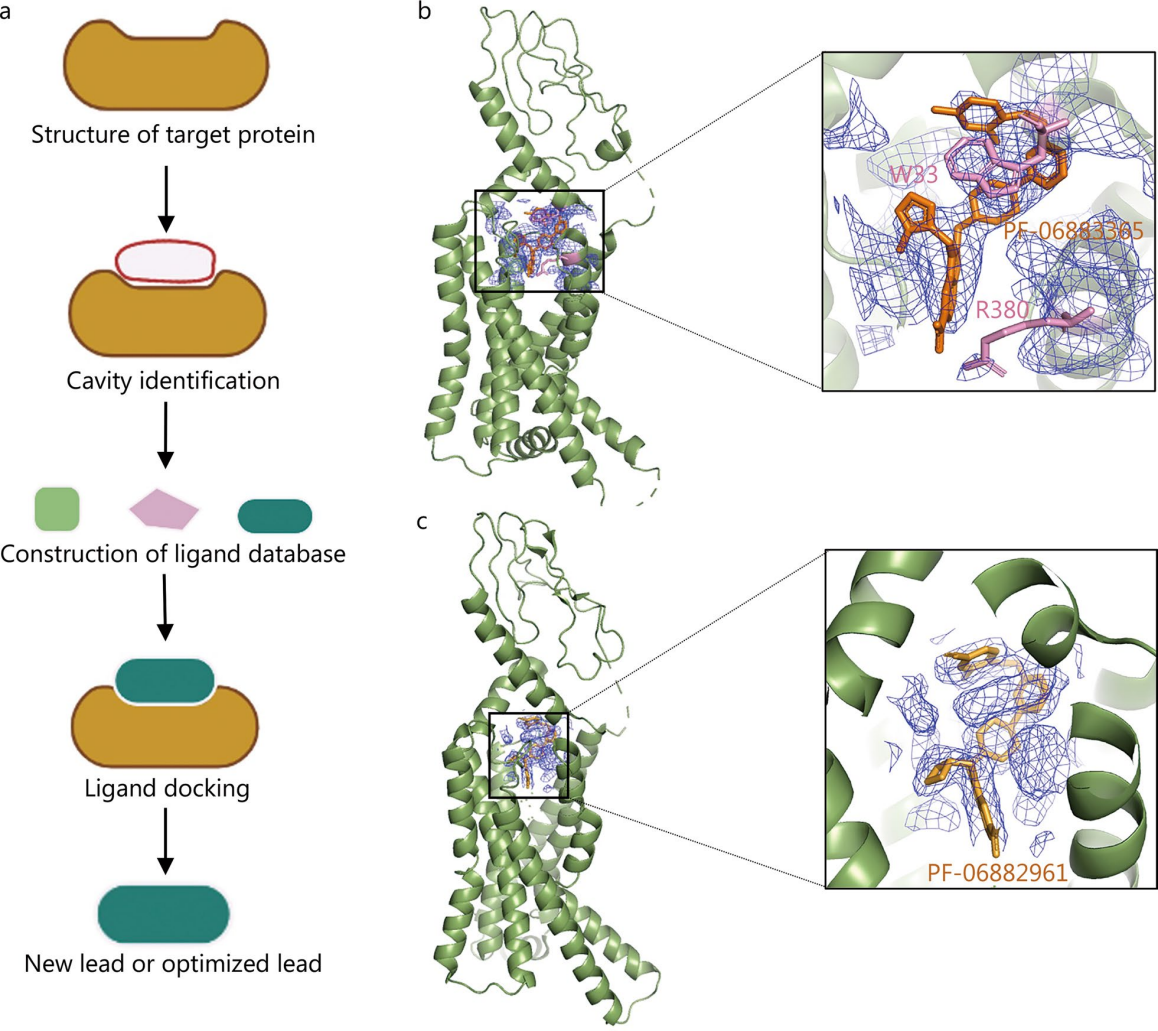
Application of cryo-EM in structure-based drug design (SBDD). **a** Schematic diagram of SBDD technology. **b**, **c** Cartoon display of cryo-EM structure of GLP-1R bound with small molecule PF-06883365 and PF-06882961 at 3.8 Å and 2.5 Å resolution, respectively (PDB: 7S15. EMDB: EMD-24794 for PF-06883365 bound state. PDB: 6X1A. EMDB: EMD-21994 for PF-06882961 bound state). The panel shows the map density of the ligand and the key residues for the binding of the agonist. GLP-1R glucagon-like peptide-1 receptor

SBDD is a streamlined drug design method based on molecular recognition of the three-dimensional structure of ligands and target proteins, with the goal of finding and optimizing small-molecule drugs. Usually, the steps of SBDD include structure determination of the target protein, cavity identification, ligand database construction, ligand docking and lead discovery (Fig. 2a) [36]. SBDD is the current mainstream mode of drug development. It is usually combined with a computational-based fast virtual screening of large chemical libraries, which lowers the cost of the initial screenings. It can directly analyze the binding energy between drugs and targets, improve the hit rate of drug discovery, and assist rational drug design. Many drugs have reached the market through SBDD technology, such as captopril, an angiotensin-converting enzyme (ACE) inhibitor developed by Bristol Myers Squibb [37], which was the first to use enzyme-inhibitor structural information; saquinavir, which targets the human immunodeficiency virus protease, developed by Roche [38]; zanamivir, which is the neuraminidase inhibitor developed by Biota [39, 40]; and the most famous breakpoint cluster region-proto-oncogene tyrosine-protein kinase inhibitor, imatinib [41], developed by Novartis for the treatment of leukaemia. However, the shortcomings of SBDD are also obvious, and obtaining high-resolution and complete structural information on protein targets is a prerequisite for SBDD. Most of the target structures in SBDD are provided by X-ray crystallography. However, for membrane proteins and macromolecular complexes, obtaining high-quality crystal structures is particularly difficult [42, 43]. With the rapid development of cryo-EM, this difficulty is being gradually resolved. Taking G-protein-coupled receptors (GPCRs) as an example, cryo-EM has shown incomparable effectiveness in SBDD [44].

GPCRs are the most abundant receptor membrane proteins on the cell surface and are targeted by approximately 30% of drugs on the market, including agonists and antagonists of GPCRs [45]. Although structural information of the GPCR targets for many marketed drugs has been obtained by X-ray crystallography, the highly dynamic nature of GPCRs makes it difficult to obtain high-quality crystals, so X-ray crystallography diffraction is powerful but challenging for many GPCR family members [46]. The natural advantages of cryo-EM for the structural elucidation of membrane proteins and highly dynamic proteins have facilitated the development of drugs targeting GPCRs [44, 47–49]. Taking glucagon-like peptide-1 receptor (GLP-1R) protein as an example, cryo-EM has played an important role in the development of oral small molecule agonists of GLP-1R [50]. GLP-1R is a member of the class B GPCR family. The binding of GLP-1 peptide to its receptor GLP-1R can activate GLP-1R to promote the proliferation of pancreatic β cells and increase insulin levels. Therefore, GLP-1R is one of the most effective targets for the treatment of type 2 diabetes [51]. GLP analogues such as exenatide, liraglutide, and semaglutide have been used in the clinical treatment of diabetes and obesity [52]. However, most of these peptide analogues are injection-based. In 2017, to rationally design small-molecule agonists that can be used orally, Zhang et al. [53] reported the crystal structure of GLP-1R binding to negative allosteric modulators, which provided structural information for GLP-1R drug development. However, it is difficult to obtain the activated conformation for crystallization and thus the binding site of the activator cannot be determined using X-ray crystallography. In 2020, Griffith et al. [54] improved the high-throughput screening of GLP-1R small-molecule agonists, carried out a series of optimizations to obtain lead compounds through structure–activity relationship, and finally obtained the small molecule agonist PF-06882961, which has high oral availability (Fig. 2b). At the same time, they analysed the electron microscopic structure of the complex of PF-06883365, an analogue of PF-06882961, with GLP-1R, revealed the interaction mechanism between the small-molecule agonists and GLP-1R, and identified the key factors of W33 and R380 in the activation process (Fig. 2c). This study laid a foundation for the development of GLP-1R-based SBDD drugs and the optimization of lead compounds.

Additional examples for the application of cryo-EM in SBDD can be found in ion channels such as Transient Receptor Potential Cation Channel Subfamily A Member 1 (TRPA1) [55] and Transient Receptor Potential cation channel subfamily V member 1 [56], gamma-aminobutyric acid receptor [57, 58], insulin receptor [59, 60], as well as biased GPCR ligands like GLP-1 receptor-Gs complex [61], mu-opioid receptor [62], non-covalent TRPA1-biased agonist GNE551 [63, 64] and sphingosine-1-phosphate receptor 1 [65].

## Application of cryo-EM in FBDD

**Fig. 3:**
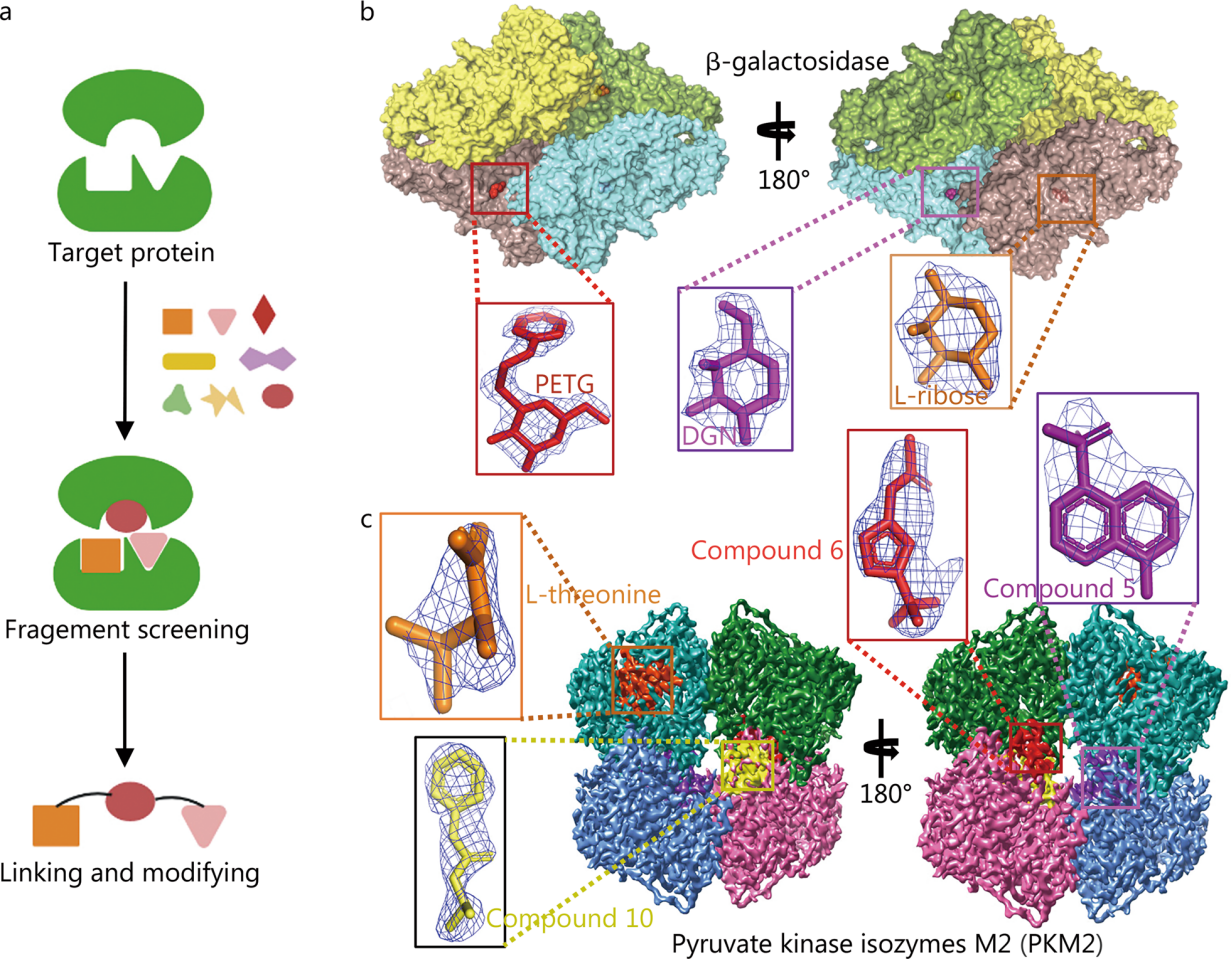
Application of cryo-EM in fragment-based drug design (FBDD). **a** Schematic diagram of FBDD technology. **b** Cryo-EM 3D map of β-galactosidase (Bgal) colored by chain. The active sites are marked by black box and displayed in different color. Structures of β-D-thiogalactopyranoside (PETG), L-ribose and 1-deoxy-glactonojirimycin (DGN) fit to the density maps of the Bgal complexes (PDB: 6TTE, 6TSK, 6TSH. EMDB: EMD-10574, EMD-10564, EMD-10563). The molecules are colored differently according to the active sites, respectively. **c** Cryo-EM 3D map of pyruvate kinase isozymes M2 (PKM2) colored by chain. The compound binding sites are marked by box and displayed in different color. Structures of L-theronine, compound 5, compound 6 and compound 10 fit to the density maps of the PKM2 complexes (PDB: 6TTH, 6TTF, 6TTI, 6TTQ. EMDB: EMD-10576, EMD-10575, EMD-10577, EMD-10584). The molecules are colored differently according to the cavities

FBDD is one of the mainstream methods for lead compound discovery and involves obtaining structural information on the target, fragment screening, and fragment linking and modification, as shown in Fig. 3a [66]. Nuclear magnetic resonance, surface plasmon resonance, and other technologies can be used to screen for small-molecule fragments that have weak interactions with target proteins and then to optimize and connect active fragments based on their structural information to design lead compounds with higher activity. Unlike high-throughput screening to find macromolecules that fit multiple active pockets at the same time, FBDD aims to find small fragments that fit a single active pocket. It requires a small compound library and has a high tolerance for pocket depth, even for some near-flat protein interaction pockets. FBDD technology also has a high success rate. However, the application of FBDD technology relies critically on the three-dimensional structural information of the target protein, for which purified protein is essential [67]. Similar to SBDD, the acquisition of a three-dimensional structure is usually the main factor limiting the development of FBDD technology. FBDD technology is in principle suitable mainly for proteins with multiple active sites and high molecular mass. The structural elucidation of suitable targets for FBDD using traditional crystal diffraction techniques is challenging. Therefore, although FBDD has been in development for more than 20 years, only six FBDD drugs, vemurafenib, venetoclax, erdafitinib, pexidartinib, sotorasib and asciminib, have been approved for the market [68–70].

Cryo-EM has obvious advantages for the structural analysis of macromolecular proteins, and the resolution level is the main consideration for whether cryo-EM can contribute to FBDD. In 2020, Yip et al. [34] and Nakane et al. [35] independently reported the cryo-EM structure of apoferritin at 1.2 Å resolution, marking the development of this technology to a real atomic resolution level. Subsequently, Saur et al. [71] first explored the application of cryo-EM to FBDD with the β-galactosidase (Bgal) system and the cancer-related kinase pyruvate kinase isozymes M2 (PKM2) system as pilot studies. Bgal catalyses the hydrolysis of lactose to galactose and glucose [72]. The cryo-EM structure of Bgal shows that the protein has two deep binding pockets, which can bind L-ribose and 1-deoxy-glactonojirimycin and are closed by a loop between G794-P803, in addition to a shallower pocket that can bind compounds such as β-D-thiogalactopyranoside (Fig. 3b). Saur et al. [71] used cryo-EM to analyse the 2.2–2.3 Å resolution complex structure of the above three small molecules, which clearly displayed the small-molecule structure of the binding site and the conformational changes of the active site protein, indicating that cryo-EM can be used to guide FBDD drug discovery (Fig. 3b). Next, they screened the small-molecule fragments of PKM2 [71], which is a target kinase in cancer development and catalyses the conversion of phosphoenolpyruvate to pyruvate [73]. After structural analysis, the 2.6–3.2 Å complex structures of multiple compounds with PKM2 were determined, and the applicability of cryo-EM in FBDD was further demonstrated (Fig. 3c) [71].

## Application of cryo-EM in PROTAC

**Fig. 4:**
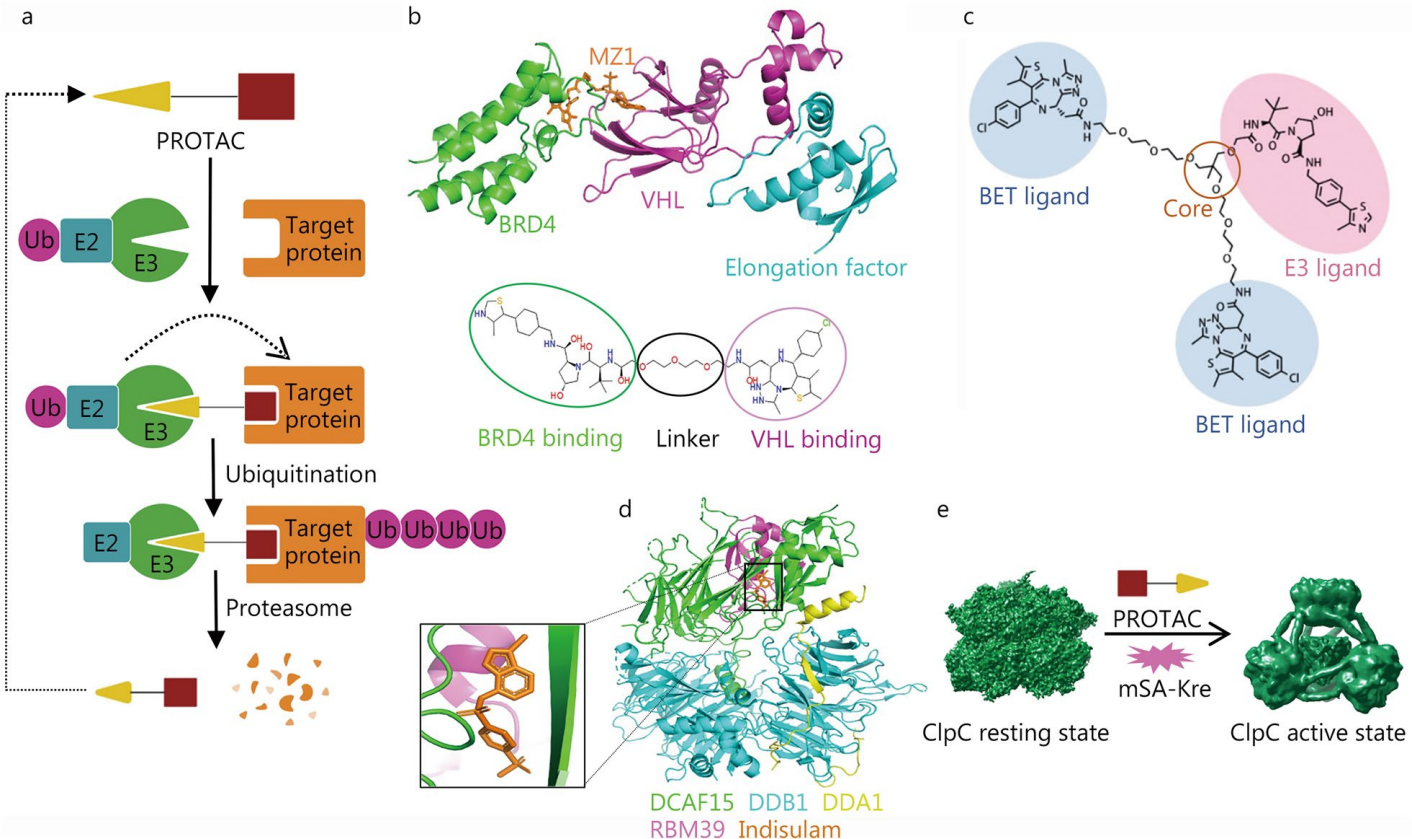
Application of cryo-EM in proteolysis targeting chimera (PROTAC). **a** Schematic diagram of PROTAC technology. **b** Cartoon display of the MZ1 mediated BRD4-VHL complex and the design of the MZ1 (PDB: 5T35). **c** Structure of the trivalent PROTAC. **d** Cartoon display of the human DCAF15-DDB1-DDA1-RBM39 complex with indisulam (PDB: 6SJ7. EMDB: EMD-10213). **e** BacPROTAC mediated ClpC activation for target protein degradation (EMDB: EMD-11707, EMD-11708). PROTAC proteolysis targeting chimera, BRD4 bromodomain-containing protein 4, VHL Von Hippel-Lindau, Ub ubiquitin, E2/3 ubiquitin protein ligases 2/3, BET bromodomain and extra-terminal domain, DCAF15 DDB1 and CUL4 associated factor 15, DDB1 DNA damage-binding protein 1, DDA1 DET1 and DDB1 associated 1, RBM39 RNA-binding motif protein 39, ClpC AAA^+^ unfoldase, mSA-Kre monomeric streptavidin fused Kre protein

Most small-molecule drugs need to bind to the active site or pocket of the target protein to function. However, for proteins that do not have suitable surface pockets, those drugs are powerless as they cannot bind to the target properly [74]. PROTAC is a drug development technology that utilizes the ubiquitin–proteasome system to degrade target proteins [75]. The PROTAC drug resembles a dumbbell structure and consists of three parts, an E3 ubiquitin ligase ligand, a target protein–ligand, and a special linker that connects the two active ligands. When the PROTAC drug enters the patient’s body, the target protein–ligand and the E3 ubiquitin ligase ligand bind to the corresponding protein, thereby recruiting the E3 ubiquitin ligase to the vicinity of the target protein and ubiquitinating the target protein, resulting in the degradation of the target protein by proteases (Fig. 4a) [76]. PROTAC technology not only can access some difficult accessible drug binding sites but also has the advantages of small doses, high selectivity and the ability to overcome drug resistance as it can eliminate the overexpressed or mutated targets [77, 78].

The design of both active ligands and intermediate linkers of PROTAC is highly dependent on the structural information of the acting complex. MZ1 was the first PROTAC drug developed, with active ligands at both ends linked to the ubiquitin ligase von Hippel–Lindau (VHL) and the bromodomain and extra-terminal domain family protein bromodomain-containing protein 4 (BRD4) for cancer treatment (Fig. 4b) [78]. In addition, a study has shown that the small-molecule inhibitor MT1 can bind to BRD4, so based on the crystal structure of VHL-MZ1-BRD4, a new trivalent PROTAC drug has been developed that increased the effectiveness by 300-fold (Fig. 4c) [79], showing the importance of structural information in PROTAC drug development. Novartis combined X-ray crystallography and cryo-EM to resolve the anticancer mechanism of indisulam [80]. These studies showed that indisulam functions as a PROTAC drug and induces the degradation of mRNA spliceosome RNA-binding motif protein 39 (RBM39) by promoting the interaction between the E3 ubiquitin ligase DNA damage-binding protein 1 and cullin 4 associated factor 15 and RBM39, leading to cancer cell death (Fig. 4d). Although the electron microscope structure shows that indisulam specifically binds to RBM39, this approach is difficult to use for targeted therapy at other sites. However, the example of trivalent MZ1 shows that the structure-based modification of PROTAC drugs may expand the target range and improve target degradation.

In addition to cancer treatment, PROTAC drugs are promising to treat neurodegenerative diseases [81, 82], antiviral infection [83, 84], and antibacterial therapy [85]. For the treatment of neurodegenerative diseases, the main PROTAC targets are Tau protein and α-synuclein [86, 87]. Peptide-based α-synuclein degraders have been experimentally shown to protect neurons from neurotoxicity induced by α-synuclein overexpression. The feasibility of antiviral PROTAC drugs has also been established in the study that degrade hepatitis C virus nonstructural protein 3/4A protease [88]. With the prevalence of the coronavirus disease 2019 (COVID-19), PROTAC technology has great potential for the targeted treatment of COVID-19 infection. The main proteases main protease and papain-like protease of the new coronavirus and RNA-dependent RNA polymerase are potential targets of PROTAC molecules [89]. Recently, Morreale et al. [85] reported a BacPROTAC drug molecule and firstly explored the application of PROTACs in antibacterial activity, with analysis of the cryo-EM structure of AAA^+^ unfoldase:caseinolytic protease P (ClpC:ClpP) in the apo state and drug binding state. ClpC:ClpP protease exists in gram-positive bacteria and recognizes phosphorylated arginine residues, which act as a degradation signal similar to the ubiquitin system in eukaryotic cells. The well-designed BacPROTAC molecule can bind to both the target protein monomeric streptavidin fused Kre and the ClpC:ClpP protease, and activate ClpC for target protein degradation (Fig. 4e).

## Application of cryo-EM in antibody drug development

**Fig. 5:**
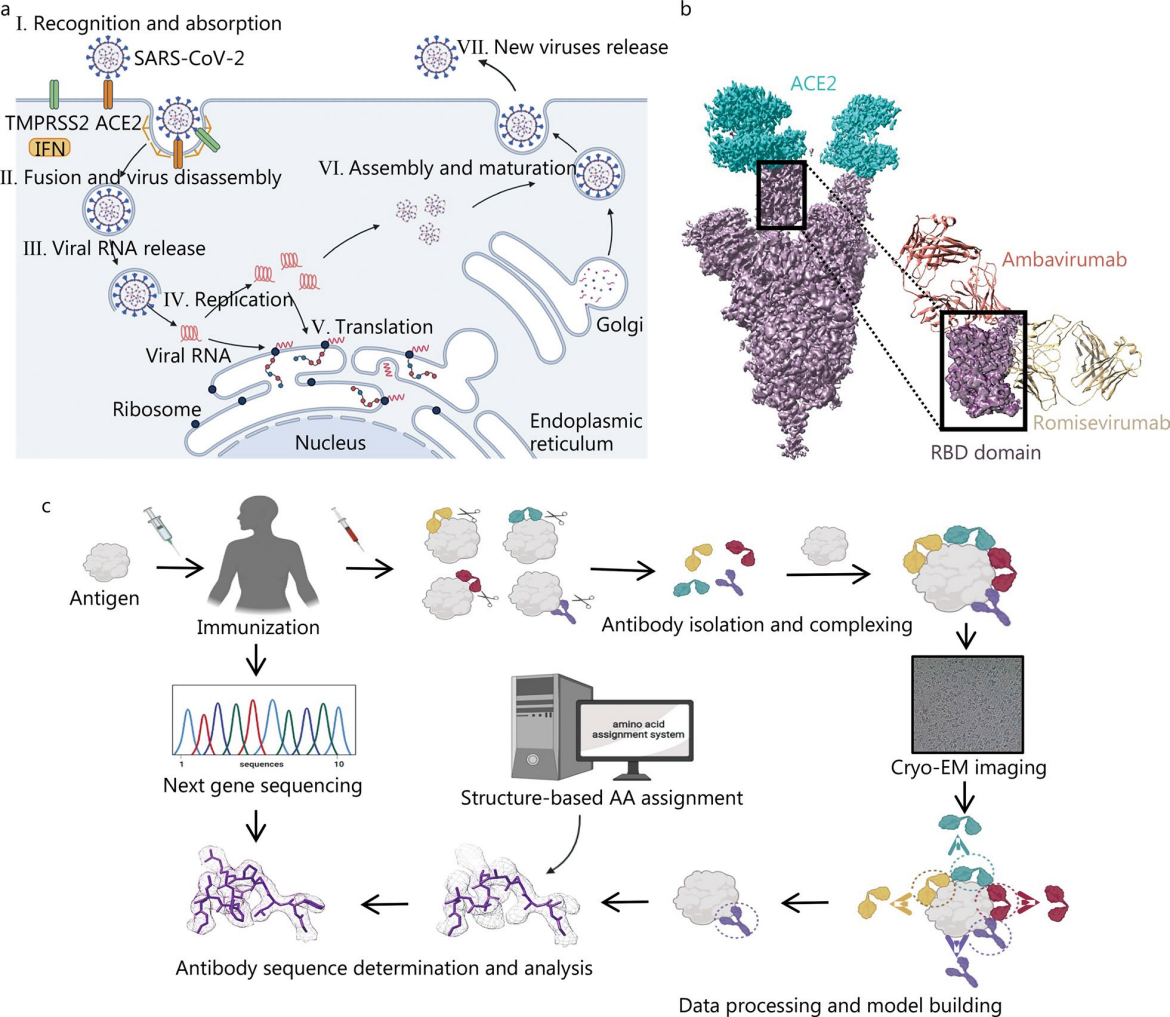
Application of cryo-EM in antibody drug development. **a** Replication cycle of SARS-CoV-2. **b** Structure of S-ACE2 complex associates with the designation of ambavirumab and romisevirumab (EMDB: EMD-25760). **c** Schematic diagram of cryo-EM and sequencing based antibody discovery. SARS-CoV2 severe acute respiratory syndrome coronavirus 2, ACE2 angiotensin-converting enzyme 2, TMPRESS2 Transmembrane serine protease 2, IFN type I interferon, RBD receptor-binding domain, RNA ribonucleic acid

COVID-19 has a major impact on the health of human society. It is caused by a virus named SARS-CoV-2. Cryo-EM has played an important role in the research and development of novel antibodies and small molecule therapeutics targeting SARS-CoV-2 infection [90]. Viral infection of human cells usually includes the following consecutive steps: I. recognition and absorption of the virus, II. virus fusion and disassembly, III. viral RNA release, IV. replication, V. translation, VI. assembly and maturation, VII. new virus release (Fig. 5a) [91]. Antibody drugs mainly work by blocking the adsorption and invasion of human cells by SARS-CoV-2. SARS-CoV-2 induces the expression of human angiotensin-converting enzyme 2 (ACE2) by producing interferon, which acts as a viral receptor to bind to the virus surface S protein and induce the transmembrane serine protease 2 (TMPRSS2) to mediate the rearrangement of the S protein, causing the fusion of the virus and the host cell plasma membrane. Therefore, the structural analysis of the S protein and ACE2 protein complex reveals important drug target sites and provides support for the elucidation of the immune escape mechanism of mutant strains and the development of antibody drugs [92].

Ambavirumab/Romisevirumab combination therapy is currently the only antibody drug approved by the State Food and Drug Administration of China. The two antibodies target different epitopes that bind to the S protein receptor and maintain therapeutic activity against a variety of mutant strains, including Omicron (Fig. 5b) [93–96]. The efficacy of the two antibodies is enhanced without competition and can effectively block the invasion of the infection of SARS-CoV-2. Among them, ambavirumab acts on the direct binding site of the spike (S) protein and ACE2, blocking the binding of the virus to ACE2, while romisevirumab acts on receptor binding site IV of the S protein to inhibit the fusion of the virus and the cell membrane. The structural information on the S-ACE2 complex and S-antibody complex obtained by cryo-EM provides important guidance for developing antibody-based drugs such as antibody screening, elucidating the mechanism of action, and structure optimization [96].

The acquisition of monoclonal neutralizing antibodies for the treatment of COVID-19 is usually obtained by screening and separation from the plasma of patients who have recovered after being infected by the virus [97]. Shortening the screening and structure optimization time of highly efficient antibodies is a long-term goal of antibody development strategies. A recent study reported that the combination of cryo-EM and next-generation sequencing can quickly obtain key epitope information without isolating monoclonal antibodies, greatly shortening the time for antibody development [98]. The main process is shown in Fig. 5c. After immunizing animals or humans with labelled antigens to obtain antigen and polyclonal antibody complexes, cryo-EM is used to analyse the complex structure to confirm key epitopes and build epitope models. Considering the complexity of the manual matching process between density maps and amino acids, they also developed an algorithm tool for identifying antibody sequences based on density maps and matched and scored them with the antigen-binding specific B-cell next-generation sequencing database, thus quickly obtaining epitope information and monoclonal antibody structural models. Recently, the structures of B-cell receptors have also been resolved by cryo-EM, which promotes the development of antibody-based therapeutics [99, 100]. In conclusion, cryo-EM can guide and promote antibody drug screening, epitope research, and engineering transformation and is an effective tool for human health research.

## Application of cryo-EM in drug failure and drug repurposing

**Fig. 6:**
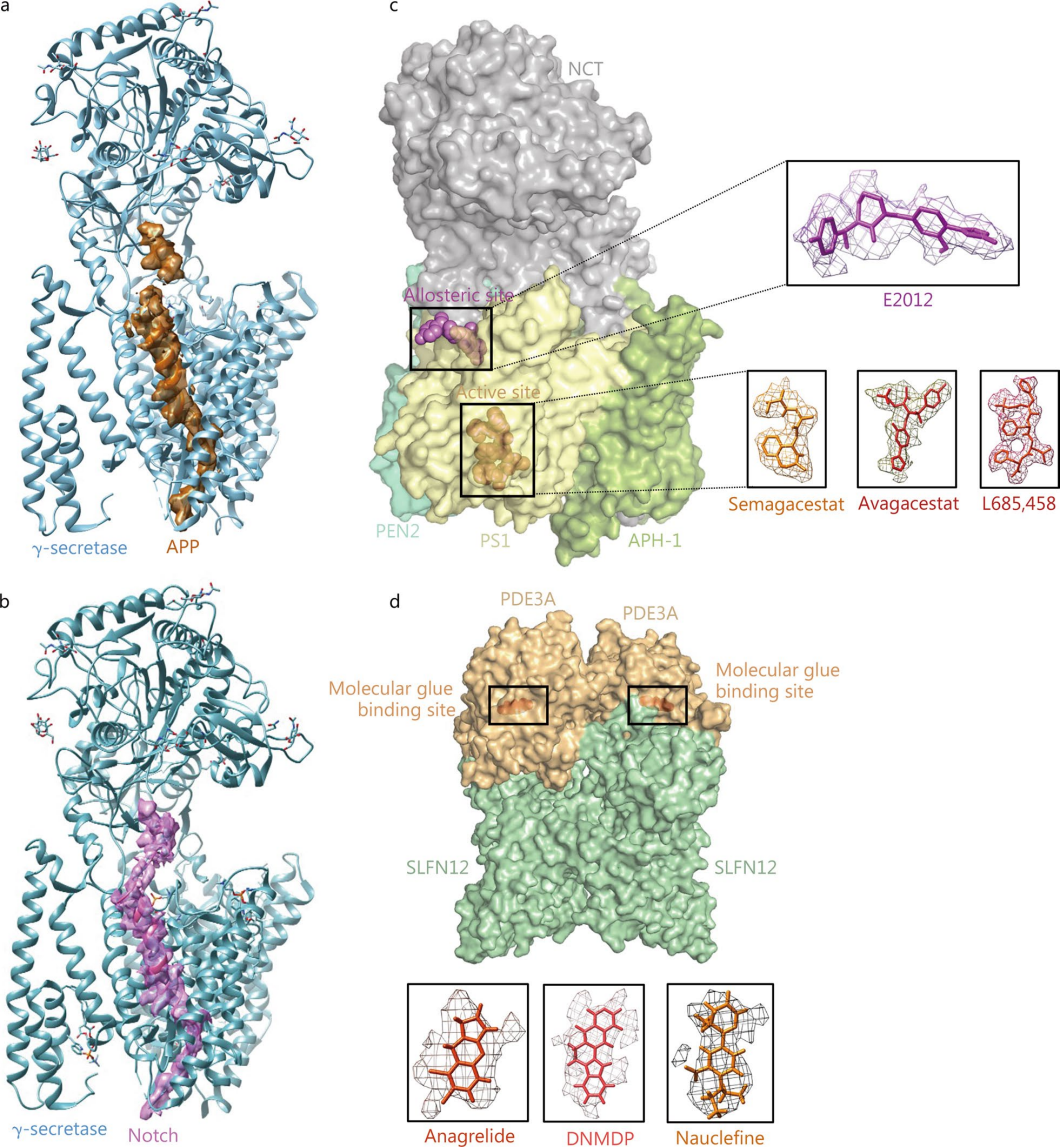
Application of cryo-EM in drug repurposing and drug failure. **a**, **b** Structures of γ-secretase bound to its substrates (PDB: 6IYC, 6IDF. EMDB: EMD-9751, EMD-9648). **c** Modulators and inhibitors of γ-secretase bound to allosteric and active sites (PDB: 6LR4, 6LQG, 7C9I, 7D8X. EMDB: EMD-0957, EMD-0944, EMD-30312, EMD-30614). **d** Molecular glues mediate the interaction of PDE3A and SLFN12 (PDB: 7EG0, 7EG1, 7EG4. EMDB: EMD-31103, EMD-31104, EMD-31105). APP amyloid precursor protein, NCT nicastrin, PEN2 presenilin enhancer 2, PS1 presenilin 1, APH-1 anterior pharynx-defective 1, PDE3A phosphodiesterase 3A, SLFN12 schlafen protein family 12, DNMDP 6-[4-(Diethylamino)-3-nitrophenyl]-5-methyl-4,5-dihydropyridazin-3(2H)-one

Drug discovery is full of uncertainty. Most of the lead compounds or small-molecules obtained through high-throughput screening may be failed due to side effects. By revealing the mechanism of target-drug interactions, cryo-EM can reveal the potential reasons of drug failure or side effects [101]. For example, γ-secretase is considered to be an important therapeutic target for Alzheimer’s disease. Although a variety of small-molecule drugs targeting γ-secretase have been developed through high-throughput screening techniques, many of them have failed due to serious side effects. Yang et al. [102] reported the cryo-EM structures of γ-secretase in the substrate binding states [amyloid precursor protein (APP) and Notch, respectively] and 4 inhibitor binding states (semagacestat, avagacestat, L685,458 in active site and E2012 in allosteric site) (Fig. 6a–c) [103]. Structural analysis showed that the main reason for the failure to develop γ-secretase inhibitors is that γ-secretase inhibitors lack specificity or have weak selectivity for APP and Notch proteolysis, thus cannot specifically inhibit APP binding [103]. It is worth noting that although the development of several small-molecule drugs for the treatment of Alzheimer’s has failed, small-molecule drugs such as nirogacestat have shown good efficacy against invasive fibroids, providing an example of drug repurposing [104]. Nirogacestat’s anti-tumour effect is precisely its side effect of inhibiting Notch hydrolysis in Alzheimer’s treatment, which enables a new application of the old drug. In fact, cryo-EM has now become a reliable tool for drug repurposing. Based on the PRISM drug reuse resource library developed by Corsello et al. [105], Chen et al. [106] used cryo-EM to investigate the potential value of three small-molecule compounds {anagrelide, nauclefine, and 6-[4-(Diethylamino)-3-nitrophenyl]-5-methyl-4,5-dihydropyridazin-3(2H)-one} as tumour suppressor drugs. They analysed the complex structures of phosphodiesterase 3A (PDE3A) and Schlafen protein family (SLFN) member SLFN12 with the three small molecules and found that these three small-molecule drugs can promote the complex formation of the two proteins and induce cellular apoptosis (Fig. 6d) [106]. Based on comprehensive considerations such as pharmacokinetics and safety, they finally modified anagrelide, which was originally used for the treatment of thrombocytosis, and obtained tumour inhibition effects in animal experiments.

## Combination of cryo-EM and artificial intelligence

AI is a broad interdisciplinary term generally referring to the ability of a computer to perform tasks commonly associated with human beings, which is learning and problem-solving, rooted in logic, statistics, neuroscience and computer engineering [107]. AI and its subdiscipline machine learning (ML) are now playing increasingly essential roles in health care-related fields such as drug discovery [108, 109]. AI techniques, especially ML, could contribute to almost every aspect of drug discovery, including the identification of new drug targets, computer-aided drug screening, synthetic accessibility, and retrosynthesis predictions that aid synthesis planning as well as various ML-based methods of drug toxicity prediction [110–113]. Most importantly, AI-based protein structure prediction methods such as AlphaFold2 are expected to have a dramatic impact on drug discovery by aiding the structure determination and molecular mechanism analysis of human diseases [114]. Lupas et al. [115] have reported the successful application of AlphaFold2 to the crystal structure determination of an archaeal transmembrane receptor, which they had failed to solve for almost a decade because phasing problems and the lack of a template hindered the performance of molecular replacement, although they had diffraction data with good quality. The AlphaFold2-produced template solved the problem with extremely high accuracy. Recently, Fontana et al. [116] reported the structure of the cytoplasmic ring of the nucleus pore complex by integrative cryo-EM and AlphaFold. The nuclear pore complex (NPC) is the molecular conduit located in the nuclear membrane of eukaryotic cells that mediates the exchange of biomolecules between the nucleus and the cytosol. To better understand the organization, dynamics and complexity of NPCs, they reconstructed the cytoplasmic ring map of *Xenopus laevis* NPC at 6–7 Å resolution and used AlphaFold to predict the structures of the individual subunits and the complex structures to guide model fitting of the CR map. This case gives an example of integrative cryo-EM and AlphaFold as a general tool to obtain more sophisticated models from medium-resolution density maps. In addition, AI has facilitated the advance of cryo-EM data collection [117] and data processing [118, 119]. With the technique of reinforcement learning (RL), the algorithm cryoRL has been used to solve the path planning problem to maximize data quality and minimize human intervention during data collection [117]. Topaz is an efficient and accurate particle-picking program based on neural networks and ML that has shown merits in conventionally challenged proteins with small, nonglobal and asymmetric particles and improving the signal-to-noise ratio in cryo-EM image processing [118, 119]. Currently, the automation, throughput and resolution level are the predominant obstacles restricting the application of cryo-EM in drug development [120]. AI might be the key to solving these problems, and the integration of cryo-EM and AI techniques promise to substantially accelerate drug development.

## Conclusions

Structural biology has always played an important guiding role in drug development. With the development of new techniques, the role of cryo-EM in drug development strategies such as SBDD, FBDD, and PROTAC has become increasingly important. However, it cannot be ignored that cryo-EM still has certain limitations in drug research and development, mainly in the following three aspects. Firstly, cryo-EM still presents certain technical barriers in the analysis of small proteins but there are many efforts taken which include the introduction of new electron optical apparatuses like energy filter, Cs-corrector, and Volta phase plate, as well as the utilization of scaffolds which increases the molecular weight of the target protein. Lys-Asp-Glu-Leu receptor, which is about 23 kD, is the smallest membrane protein resolved by cryo-EM with sufficient map density for de novo model building [19]. Second, the resolution of cryo-EM still needs improvement. Although the current highest resolution reached 1.2 Å, most of the reported cryo-EM structures are still at the 3–4 Å level, and therefore the structural information on drug molecule binding target sites usually needs to be combined with higher resolution protein structures obtained from X-ray crystallography. Finally, cryo-EM data collection and processing lag a bit behind X-ray crystallographic diffraction. But it is worth noting that the situation is getting better and there are now dramatic improvements in both automated data collection using programs such as serialEM [121], multigrid cartridges in state of the art microscopes, as well as on the fly data processing such as cryoSPARC live [122]. Modern AI techniques may help overcome those limitations of cryo-EM in drug development in the near future.

## Data Availability

Not applicable.

## Abbreviations

ACE2: Angiotensin-converting enzyme 2
AI: Artificial intelligence
APP: Amyloid precursor protein
Bgal: β-Galactosidase
BRD4: Bromodomain-containing protein 4
ClpC: AAA^+^ unfoldase
ClpP: Caseinolytic protease P
COVID-19: Coronavirus disease 2019
Cryo-EM: Cryo-electron microscopy
FBDD: Fragment-based drug discovery
GLP-1R: Glucagon-like peptide-1 receptor
GPCR: G-protein-coupled receptor
ML: Machine learning
NMR: Nuclear magnetic resonance
NPC: Nuclear pore complex
PKM2: Pyruvate kinase isozymes M2
PROTAC: Proteolysis targeting chimera
RBM39: RNA-binding motif protein 39
RL: Reinforcement learning
SARS-CoV-2: Severe acute respiratory syndrome coronavirus 2
SBDD: Structure-based drug design
SLFN: Schlafen protein family
VHL: Von Hippel–Lindau
